# The value of repeated debridement, antibiotics, and implant retention (DAIR) for early periprosthetic joint infection

**DOI:** 10.5194/jbji-10-207-2025

**Published:** 2025-07-10

**Authors:** Ruben Scholten, Gerjon Hannink, Matthijs P. Somford, Job L. C. van Susante

**Affiliations:** 1 Department of Orthopedics, Rijnstate Ziekenhuis, Wagnerlaan 55, 6815 AD Arnhem, the Netherlands; 2 Department of Operating Rooms, Radboud University Medical Center, P.O. Box 9101, 6500 HB Nijmegen, the Netherlands

## Abstract

**Background and purpose**: Debridement, antibiotics, and implant retention (DAIR) is the proposed initial treatment of early periprosthetic joint infection (PJI), but it may fail to provide infection control. Subsequently, either implant removal or repeated DAIR may be considered. This study aims to identify the failure rate of repeated DAIR for early PJI in primary total knee arthroplasty (TKA) and total hip arthroplasty (THA). **Methods:** All DAIRs performed following primary THA or TKA for early PJI from 2010 to 2019 were retrospectively analysed. Patient demographics, comorbidities, surgical details, and pre-DAIR C-reactive protein (CRP) levels were recorded. Failure of early infection control (within 1 month after DAIR) prompted a second DAIR. Follow-up was performed up to 2 years post-surgery. A Kaplan–Meier survival analysis was performed in single- and repeated-DAIR groups. Cox regression analyses explored potential risk factors for implant failure after repeated DAIR. **Results:** A total of 124 cases of early PJI were included. Single DAIR achieved adequate infection control in 69.4 % ( n=86 ) of cases, while 30.6 % ( n=38 ) of cases underwent repeated DAIR within 3–23 d. After 2 years, implant removal was performed in 8 cases (9.9 %; 95 %CI 3.0 %–16.0 %) in the single-DAIR group and in 8 cases (22.2 %; 95 %CI 7.3 %–34.7 %) in the repeated-DAIR group. No statistically significant associations between the failure of repeated DAIR and its potential risk factors were found. **Conclusion:** If initial DAIR does not achieve early PJI control, repeated DAIR can still be considered, as it may avoid implant removal in 77.8 % of cases. The authors advocate for tailored decisions considering implant revisability, patient comorbidity, and pathogen susceptibility.

## 1 Introduction

Periprosthetic joint infection (PJI) is a severe complication following total joint arthroplasty (TJA). For early PJI (occurring within 3 months of implantation), the standard treatment is debridement, antibiotics, and implant retention (DAIR) (Osmon et al., 2013). Successful DAIR is defined by implant retention without infection signs after 12 weeks of antibiotics, with success rates of between 26 % and 92 % reported. (Azzam et al., 2010; Kurtz et al., 2018; Tsukayama et al., 1996; Fehring et al., 2013; Qasim et al., 2017; Zhang et al., 2020; Kuiper et al., 2013; Bradbury et al., 2009; Qu et al., 2019).

DAIR failures can be categorized as early failures (where infection control is not achieved) or late failures (where infection relapses after initial success) (Sousa and Abreu, 2018). Late failures typically involve implant loosening or bone loss, with associated symptoms like joint pain and elevated inflammatory markers. Early failures manifest through signs of progressive infection (i.e. wound breakdown, wound erythema, local pain and/or swelling, rising levels of C-reactive protein (CRP), fever, or even sepsis). This study addresses treatment options if early DAIR fails to achieve infection control. In this case, the surgeons must decide whether to repeat DAIR or proceed with implant removal and revision surgery. While a repeated DAIR may seem appealing (as it omits the need for extensive revision surgery), its success rates are debatable (Argenson et al., 2019). Most studies report on the outcome of single DAIR procedures, whereas data on repeated DAIR procedures are limited with respect to number and clarity; moreover, studies commonly mix early- and late-PJI cases (Vilchez et al., 2011; Lora-Tamayo et al., 2013; Urish et al., 2018; Lizaur-Utrilla et al., 2015; Byren et al., 2009; Kuiper et al., 2013; Geurts et al., 2013; Mont et al., 1997; Azzam et al., 2010; Triantafyllopoulos et al., 2016). Based on the limited evidence on repeated DAIR procedures, the International Consensus on Orthopaedic Infections recommends considering revision arthroplasty following failed DAIR, as additional DAIR procedures are, at best, equally effective compared to primary DAIR (Argenson et al., 2019). However, if successful, repeated DAIR could be worthwhile due to its lower burden on patient and healthcare systems. The latter is supported by a study performed in 2020 that demonstrated that a second DAIR had a low failure rate and that, therefore, a second DAIR should not be discarded in the treatment of acute PJIs (Wouthuyzen-Bakker et al., 2020). Furthermore, a recent retrospective multicentre study demonstrated that a second DAIR can achieve an 83 % success rate in selected patients (Auñón et al., 2024).

To further elucidate this common clinical dilemma, this study aimed to (1) identify the failure rate of a repeated DAIR procedure for unsuccessful early infection control in early PJI and (2) compare these results to the failure rate of a single DAIR with adequate early infection control. Furthermore (3), an exploratory analysis on potential procedure- or patient-related factors associated with treatment failure in cases that underwent repeated DAIR procedures was performed.

## 2 Methods

All patients who underwent a DAIR procedure between January 2010 and January 2019 within 3 months after primary elective unilateral total hip arthroplasty (THA) or total knee arthroplasty (TKA) were retrospectively identified from electronic health records at a large teaching hospital, excluding culture-negative cases.

Data collected included patient age, sex, American Society of Anaesthesiologists (ASA) classification, comorbidities (e.g. diabetes mellitus, chronic renal failure, liver failure, heart failure, coronary artery disease, stroke history, or chronic obstructive pulmonary disease), body mass index (BMI), smoking status, joint age at the first DAIR, cement usage during the index procedure, tissue culture results after repeated DAIR, and pre-DAIR CRP levels (first and any repeated DAIR). Additionally, the identity of causative pathogens was recorded. Demographic data of the patient cohort are displayed in Table 1.

**Table 1:** Demographic data on the patient cohort per group. Abbreviations used in the table are as follows: SMD – standardized mean difference; SD – standard deviation; IQR – interquartile range; TKA – total knee arthroplasty; ASA – American Society of Anesthesiologists. Renal failure was classified according to the five stages of kidney disease: G1 – kidney damage with normal or increased glomerular filtration rate (GFR) ( >90 mL min^−1^/1.73 m^2^); G2 – mild reduction in GFR (60–89 mL min^−1^/1.73 m^2^); G3a – moderate reduction in GFR (45–59 mL min^−1^/1.73 m^2^); G3b – moderate reduction in GFR (30–44 mL min^−1^/1.73 m^2^); G4 – severe reduction in GFR (15–29 mL min^−1^/1.73 m^2^); G5 – kidney failure (GFR <15 mL min^−1^/1.73 m^2^ or dialysis). Chronic obstructive pulmonary disease (COPD) was classified according to the Global Initiative for Chronic Obstructive Lung Disease (GOLD) criteria: GOLD Stage I – FEV1 ≥80 % predicted (where FEV represents forced expiratory volume); GOLD Stage II – FEV1 ≥50 % predicted but <80 % predicted; GOLD Stage III – FEV1 ≥30 % predicted but <50 % predicted; GOLD Stage IV – FEV1 <30 % predicted.

	Single-DAIR group	Repeated-DAIR group	SMD
N (number of patients)	86	38	
Age (median (IQR))	69 (64–77)	68 (61.5–77.5)	0.121
Male (%)	51 (59.3)	16 (42.1)	0.349
TKA ( %)	25 (29.1)	11 (28.9)	0.003
Cemented prosthesis	50 (58.1)	25 (65.8)	0.158
Diabetes (%)	11 (12.8)	5 (13.2)	0.011
Active smoker (%)	13 (16.2)	12 (31.6)	0.365
History of stroke (%)	7 (8.1)	4 (10.5)	0.082
Heart failure (%)	9 (10.5)	2 (5.3)	0.194
Coronary disease (%)	13 (15.1)	5 (13.2)	0.056
BMI (median (IQR))	28.09 (24.46–32.76)	28.86 (25.97–34.21)	0.222
ASA (%)			0.444
ASA1	16 (18.8)	4 (10.5)	
ASA2	53 (62.4)	20 (52.6)	
ASA3	14 (16.5)	13 (34.2)	
ASA4	2 (2.4)	1 (2.6)	
Renal failure (%)			0.298
G1	44 (51.2)	17 (44.7)	
G2	34 (39.5)	16 (42.1)	
G3a	6 (7.0)	3 (7.9)	
G3b	1 (1.2)	2 (5.3)	
G4	0 (0.0)	0 (0.0)	
G5	1 (1.2)	0 (0.0)	
COPD (%)			0.363
None	81 (94.2)	32 (84.2)	
GOLD I	2 (2.3)	4 (10.5)	
GOLD II	2 (2.3)	1 (2.6)	
GOLD III	1 (1.2)	1 (2.6)	
GOLD IV	0 (0.0)	0 (0.0)	

### 2.1 Surgical protocol

For primary THA or TKA, patients received 2 g of cefazolin prophylactically 15 to 60 min before skin incision (THA) or tourniquet inflation (TKA), followed by three doses of 1 g post-surgery at 8 h intervals (Scholten et al., 2020). THA was performed by, or under direct supervision of, senior hip surgeons. Accordingly, TKA was performed by, or under direct supervision of, senior knee surgeons. All TKA patients underwent surgery while using a tourniquet that was deflated after applying a pressure bandage over the affected knee. All TKAs were cemented and performed using a medial parapatellar arthrotomy. Both cemented and uncemented THAs were conducted using a posterolateral approach. The bone cement (PALACOS^®^ R + G; Heraeus) used in both TKA and THA contained 0.75 g of gentamicin per 61.2 g of powder.

Patients were closely monitored for post-operative infection signs and typically discharged only with a dry wound. Following discharge, all patients were subjected to protocolized surveillance of infection in the outpatient clinic for at least 3 months. In cases of wound drainage or prolonged drainage ( >10 d post-surgery), blood samples were tested for CRP, erythrocyte sedimentation rate (ESR), and leukocyte counts. For suspected superficial surgical site infections (SSI) or wound breakdown, DAIR was performed. Superficial SSI was defined according to the Infectious Diseases Society of America (IDSA) guidelines with the presence of the following: (1) purulent drainage; (2) positive culture of aseptically obtained fluid/tissue; (3) local signs and symptoms of pain or tenderness, swelling, and erythema after the incision is opened by the surgeon (unless culture-negative); or (4) SSI diagnosis by the attending surgeon (Stevens et al., 2014).

During both the first and potential second DAIR procedures, the same surgical techniques were used, and joint fluid along with six periprosthetic tissue samples were collected for culturing. Empiric antibiotic therapy (flucloxacillin, 6 g d^−1^ via continuous intravenous infusion) began after tissue cultures were obtained. All modular parts (polyethylene liner and femoral head for THAs or polyethylene insert for TKAs) were removed, followed by careful debridement and joint irrigation. Pristine modular components were then inserted. Following surgery, the patient's vitals (including temperature) were recorded every 6 h. Biochemical blood test (CRP, ESR, and leukocytes) were analysed every 2–3 d. Antibiotic treatment was adjusted based on preliminary culture results in consultation with infectiologists and microbiologists. PJI diagnosis was established according to the major Musculoskeletal Infection Society (MSIS) criteria, requiring at least two tissue cultures during DAIR showing growth of the same pathogen (Parvizi and Gehrke, 2014).

A second DAIR was performed if early infection control failed ( <1 month after initial DAIR), indicated by rising or stagnant infection markers (CRP or leucocytosis), fever, local erythema, or prolonged wound drainage ( >10 d). The instituted antibiotic treatment was continued during the second DAIR. The decision for a second DAIR was at the surgeon's discretion.

Outpatient follow-up included routine check-ups at 2 and 6 weeks and at 6 and 12 months. Regular CRP biochemical blood testing was conducted during these visits.

### 2.2 Statistical analysis

Descriptive statistics were used to summarize the data. Two distinct groups were defined: one for cases with a single DAIR and another for those with a second DAIR (repeated DAIR). Standardized mean differences (SMDs) were calculated. An SMD value of less than 0.1 indicates balance, as the SMD quantifies the difference in means between two groups relative to the pooled standard deviation. In the context of covariate balance assessment, a smaller SMD suggests that the distributions of the covariates are similar between groups. DAIR procedures were deemed a failure if any form of revision surgery (one- or two-stage exchange arthroplasty, explantation, or other surgical procedure on the joint) occurred or if suppressive antibiotic treatment was initiated.

In the repeated-DAIR group, a third DAIR procedure was also considered to be treatment failure. All patients were retrospectively analysed at least 2 years following index surgery.

A Kaplan–Meier survival analysis assessed failure rates using the aforementioned definitions for both the single- and repeated-DAIR groups. Exploratory analyses investigated associations between patient-, infection-, or procedure-related characteristics for repeated DAIR failure, employing univariate and multivariate Cox regression. Evaluated characteristics included the BMI, active smoking, polymicrobial infection, renal failure or COPD presence, implant age at first DAIR (joint age), time between first and second DAIR, bone cement usage during the index arthroplasty, and pre-DAIR CRP levels. For multivariable regression, patients with COPD and renal failure were categorized (COPD: none vs. GOLD 1 or higher; renal failure: G1 vs. G2 or higher). The proportional hazards assumption for our Cox regression model was assessed using Schoenfeld residuals. Schoenfeld tests did not indicate significant violations of the proportional hazards assumption for the models. Additionally, graphical inspection of Schoenfeld residuals over time showed no clear trends suggestive of non-proportionality. Statistical analyses were performed using R (version 4.4.0; R Foundation for Statistical Computing, Vienna, Austria).

## 3 Results

**Figure 1:**
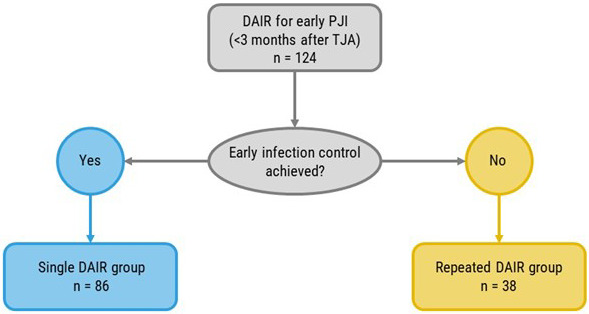
Flowchart illustrating the workflow of the study.

A total of 124 patients who underwent a DAIR procedure within 3 months after primary elective THA or TKA and who met the major MSIS criteria for (early) PJI were identified. A total of 86 (69.4 %) cases underwent a single DAIR procedure that succeeded in achieving early infection control, whereas 38 (30.6 %) patients underwent repeated DAIR due to a failure to achieve early infection control (Fig. 1). No patients went directly from initial DAIR to revision surgery within 3 months.

The median time between index surgery and the first DAIR was 20 d (range 6–79), and the median time between the first and the repeated DAIR was 11 d (range 3–23). The identified causative pathogens are displayed in Table 2. *Staphylococcus aureus* was the predominant causative pathogen involved (43.5 % of cases in both groups combined). The pathogens isolated during the repeated DAIR are displayed in Table 2.

**Table 2:** Overview of data on the involved causative pathogens per group isolated during first DAIR (single DAIR or repeated DAIR). In the third column, the data on the pathogens isolated during repeated DAIR are displayed.

	Culture results of		Culture results of
	first DAIR		repeated DAIR
	Single DAIR	Repeated DAIR	Repeated DAIR
	n=86	n=38	n=38
Coagulase-negative *Staphylococcus* (%)	17 (19.8)	8 (21.1)	6 (15.7)
*Corynebacterium* (%)	6 (7.0)	1 (2.6)	4 (10.5)
Enteric Gram-negative (%)	7 (8.1)	7 (18.4)	7 (18.4)
*Enterococcus* (%)	7 (8.1)	2 (5.3)	5 (13.1)
*Staphylococcus aureus* (%)	39 (45.3)	15 (39.5)	8 (21.1)
*Pseudomonas* (%)	0 (0)	1 (2.6)	1 (2.6)
*Streptococcus* (%)	5 (5.8)	3 (7.9)	0 (0)
Other (%)	5 (5.8)	1 (2.6)	1 (2.6)
Polymicrobial (%)	32 (37.2)	16 (42.1)	8 (21.1)
Culture-negative	–	–	15 (39.5)

**Figure 2:**
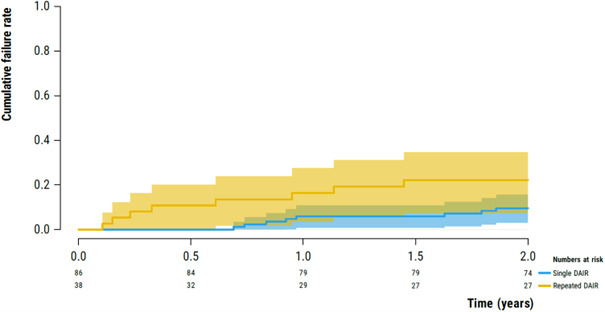
Kaplan–Meier survival analysis illustrating the failure rate of the implants following early PJI ( <3 months) after primary THA or TKA in the single-DAIR (blue) and repeated-DAIR (yellow) groups.

In the single-DAIR and repeated-DAIR groups, implant failure occurred in 8 cases (9.9 %; 95 %CI 3.0 %–16.0 %) and 8 cases (22.2 %; 95 %CI 7.3 %–34.7 %), respectively, after 2 years of follow-up (Fig. 2). No cases were identified that received suppressive antibiotics with implant retention.

Univariate Cox regression analyses did not identify any statistically significant associations between patient- or procedure-related factors and the treatment failure of repeated DAIRs (Table 3). Given the small sample size and low number of events, it was decided to not perform multivariable regression analysis.

**Table 3:** Results of the univariate and multivariable Cox regression analysis for a potential association between treatment failure and several procedure, patient, or infection characteristics, illustrated by the estimates of the hazard ratio (HR). These characteristics include the following: BMI (body mass index), active smoking by the patient, polymicrobial PJI (periprosthetic joint infection), renal failure (any degree), chronic obstructive pulmonary disease (COPD, any degree), joint age (time in days since initial arthroplasty up to the first DAIR), time interval between the first and second DAIR, cemented fixation of the implant, the CRP value prior to the first DAIR procedure, and the CRP value prior to the second DAIR procedure. The estimate is an estimation of the HR.

	Univariate Cox regression		
	HR	95 % CI	p value
BMI	1.02	0.90–1.15	0.80
Active smoker	1.33	0.32–5.58	0.69
Polymicrobial PJI	1.52	0.38–6.09	0.55
Renal failure (G1 vs. ≥ G2)	0.239	0.05–1.19	0.08
COPD (None vs. ≥ GOLD1)	0.647	0.08–5.27	0.68
Joint age	0.959	0.82–1.12	0.60
Time interval between DAIR 1 and DAIR 2	0.966	0.82–1.14	0.67
Cemented fixation	0.560	0.14–2.24	0.41
CRP prior to first DAIR	1.00	0.99–1.01	0.39
CRP prior to repeated DAIR	1.00	0.99–1.01	0.67
Total knee arthroplasty	0.71	0.14–3.45	0.68
Positive cultures during repeated DAIR	5.67	0.69–46.2	0.10

## 4 Discussion

In this study, a failure rate of 22.2 % (95 %CI 7.3 %–34.7 %) for a repeated DAIR procedure was found vs. 9.9 % (95 %CI 3.0 %–16.0 %) in a group of patients where only a single DAIR procedure was deemed necessary for the treatment of early PJI of a primary THA or TKA. These findings suggest that a repeated DAIR procedure could still be a viable treatment option in this particular context (primary THA/TKA, early PJI, and failure of early infection control), as more than 75 % of repeated DAIR procedures were able to prevent implant removal.

Repeated DAIR for early PJI treatment remains controversial. Vilchez et al. (2011) found that needing a second debridement was linked to failure of implant retention due to persistent PJI. This was supported by a large, multicentre study of *S. aureus* PJI ( n=345 ), in which a second debridement was an independent risk factor for failure (Lora-Tamayo et al., 2013). Urish et al. (2018) showed that 109 of 216 patients who underwent DAIR after TKA required additional procedures, with over 70 % of those who had repeated DAIR ultimately failing. Another study on 64 patients with early PJI ( <3 months) revealed a 61.5 % failure rate for the 39 patients who underwent DAIR, all of whom subsequently required a second DAIR without success (Lizaur-Utrilla et al., 2015).

Conversely, several studies found no association between repeated DAIR and poor outcomes (Byren et al., 2009; Kuiper et al., 2013; Geurts et al., 2013), suggesting it may be a viable option. However, available studies on repeated DAIR are limited, heterogeneous, and yield conflicting conclusions. Consequently, the international consensus on orthopaedic infections recommends considering implant removal after a failed first DAIR (Argenson et al., 2019), citing that literature generally shows a second DAIR only has an equivalent success rate compared to the initial procedure. However, following the previously mentioned consensus meeting, more favourable results of a repeated DAIR have been reported. These include the study by Wouthuyzen-Bakker et al. (2020), who demonstrated that a second DAIR had a low failure rate and that, therefore, a second DAIR should not be discarded in acute PJIs. In addition, a recent retrospective multicentre study by Auñón et al. (2024) demonstrated that a second DAIR can achieve an 83 % success rate in selected patients.

Based on both the results of the latter work and those of this study, we recognize a lower success rate in cases where early control of PJI is not achieved; however, we would like to argue against a low threshold for the removal of implants after the failure of early infection control with a single DAIR. Most of the studies underpinning the recommendation of the consensus meeting contain varying indications for DAIR (including late (hematogenous) infections and infections following revision TJA). These heterogeneous patient, procedure, and infection characteristics may have influenced treatment outcomes significantly throughout different studies, thereby explaining the wide range of reported success rates. In contrast, this study describes a relatively homogeneous cohort of patients, consisting solely of early PJI (no late or hematogenous PJI) following elective primary TKA and THA (no revision surgery). Furthermore, the DAIR procedure was standardized, including mobile bearing exchange, and a clear indication for secondary DAIR (failure of early infection control within 1 month) was defined. As such, the results of this study seem to reflect the more favourable results of a repeated DAIR encountered in the literature (Byren et al., 2009; Kuiper et al., 2013; Geurts et al., 2013; Aboltins et al., 2007; Mont et al., 1997; Auñón et al., 2024; Wouthuyzen-Bakker et al., 2020). From this perspective, repeated DAIR may yet have a role in the specific circumstances of primary TKA or THA with early PJI ( <3 months of implantation) and failure of early infection control ( <1 month after initial DAIR).

In justifying whether the chances of success may still warrant a repeated DAIR, one also has to take into account the consequences of a single-DAIR-only approach. This approach may lead to an increase in implant removals in the case of the failure of early PJI control after a single DAIR procedure. Implant removals may go hand in hand with increased patient morbidity and complications.

In our opinion, the success rate of a repeated DAIR is only part of the puzzle in clinical decision-making on whether or not to give it a chance. This study clearly indicates that the chances of successful PJI control decrease when repeated DAIR is necessary. Still, a rather low threshold towards implant removal after failed single DAIR is not always without profound consequences for the patient. Such a decision should not be made lightly. The pros and cons regarding the choice of either a repeated DAIR procedure or implant removal have to be weighed for each individual patient. Patient-specific factors like bone stock and implant fixation, antibiotic susceptibility of the responsible pathogen, options for one-stage revision, and patient comorbidities have to be taken into account.

Tools able to predict the failure of repeated DAIR would greatly aid improvement in tailored, patient-specific clinical decision-making. Previous studies have been able to identify risk factors associated with failure of initial DAIR procedures, including the time to (first) DAIR, liver cirrhosis, renal failure, use of bone cement during primary TJA, and the CRP levels prior to DAIR (Lowik et al., 2018; Izakovicova et al., 2019). These predictors would most likely also apply to a repeated DAIR procedure. In addition, few other studies have aimed to identify risk factors for the failure of a repeated DAIR. Factors identified include non-specialized surgical teams in the first DAIR, non-exchange of mobile components, polymicrobial infections, antibiotic resistance, positive cultures during the second DAIR, chronic renal insufficiency, and the time interval between DAIRs (Wouthuyzen-Bakker et al., 2020; Auñón et al., 2024; Triantafyllopoulos et al., 2016). Due to the small sample size and low number of events, it was decided to not perform multivariable regression analysis.

Our analysis is primarily exploratory, aimed at exploring hypotheses rather than drawing definitive conclusions. Based on previous studies, one should be wary of failure when considering a repeated DAIR in the case of patients with polymicrobial PJI, multiresistant pathogens, or chronic renal failure. In addition, there are recognized pathogens that are classified as more or less troublesome in the context of PJI. This distinction may influence the success rate of repeated DAIR. In the case of more resistant pathogens, such as methicillin-resistant *Staphylococcus aureus* (MRSA), *Enterococcus faecium*, and *Pseudomonas aeruginosa*, a more cautious approach may be warranted regarding repeated DAIR (Gonzalez et al., 2024; Lora-Tamayo et al., 2013). Unfortunately, this study is inadequately powered to provide specific recommendations on this topic.

There are no data to support a clear cut-off point regarding an unacceptable time interval between a first and second DAIR. However, the authors would advise caution with repeated DAIR more than a month after the first DAIR.

Future studies and potential meta-analyses, with a larger number of patients, may provide further tools to provide more specific recommendations on when to refrain from a repeated DAIR procedure in this context.

### Limitations

This study, like others on this topic, has several limitations, including its retrospective nature and relatively small cohort size, the latter of which may limit statistical analysis. It was underpowered to identify patient-, procedure-, or infection-related factors significantly associated with treatment failure. Additionally, the threshold for repeated DAIR may be lower than in other studies, potentially contributing to the relatively favourable outcomes observed. However, one could consider this consequential to our treatment strategy.

Finally, although guidelines exist for performing repeated DAIR, the decision for the go-ahead was ultimately made by various surgeons, possibly leading to differing treatment choices. However, second DAIRs are routinely performed in our hospital, and progressing with complete revision or explant surgery after DAIR is extraordinary in our practice. Still, this surgeons' discretion could certainly influence the timing and potential threshold of the second DAIR. Nonetheless, this variability may accurately reflect everyday clinical practice.

## 5 Conclusion

In a group of patients with repeated DAIR, the chances of the failure of PJI control were higher compared with patients who only required a single DAIR. Still, a success rate of more than 75 % warrants a role for repeated DAIR in the treatment of failed early PJI control ( <1 month after initial DAIR) following an initial DAIR. Tailored decision-making (considering all of the relevant factors, including implant revisability, options for one-stage revision, patient comorbidity, and pathogen antibiotic susceptibility) should be made on whether to proceed with either a repeated DAIR or revision surgery. Future studies on larger patient cohorts will potentially allow us to further discern between favourable and unfavourable patients regarding selection for repeated DAIR procedures.

## Data Availability

The data that support the findings of this study are available from the corresponding author, Job L. C. van Susante, upon reasonable request.
